# Does routine surveillance imaging after completing treatment for childhood solid tumours cause more harm than good? A systematic review and meta-analysis protocol

**DOI:** 10.1186/s13643-019-1096-3

**Published:** 2019-07-12

**Authors:** Jessica E. Morgan, Melissa Harden, Robert S. Phillips

**Affiliations:** Centre for Reviews and Dissemination, University of York, Heslington, York, YO10 5DD UK

**Keywords:** Paediatric, Cancer, Surveillance imaging, Relapse

## Abstract

**Background:**

This systemic review aims to synthesise the current literature surrounding off-therapy surveillance imaging in children and young people with extra-cranial solid tumours, with a view to establishing if routine imaging studies after treatment for childhood cancer increase overall survival, increase the psychological distress caused to patients and families, result in other harms to patients and are cost-effective strategies. Within this manuscript, we also describe how patient and public involvement has impacted upon the protocol.

**Methods:**

The search will cover thirteen different databases, key conference proceedings and trial registers, as well as reference lists and forward citations of included papers. Prominent authors/clinicians in the field will be contacted. A full search strategy is provided. The study designs to be included in the review will be added in an iterative way (RCTs, quasi-randomised trials, prospective cohorts and retrospective cohorts). Qualitative studies will also be eligible for inclusion. We will include studies which examine a programme of surveillance imaging that aims to detect relapse in children or young people up to age 25 years who have completed treatment for a malignant extracranial solid tumour and have no evidence of active and ongoing disease at end of treatment. The primary outcome is overall survival, with secondary outcomes including psychological distress indicators, number of imaging tests performed, other harms of imaging and cost-effectiveness measures. Studies will be screened and data extracted by two researchers. Studies will be critically appraised using a stratified version of the ROBINS-I tool. Where appropriate, data will be synthesised using a random effects meta-analysis. A detailed analysis plan, including assessment of heterogeneity and publication bias, is provided.

**Discussion:**

The aim of routine surveillance imaging is to detect recurrence of disease before clinical symptoms and signs develop. Some studies have suggested that most relapses of childhood cancer are detected due to clinical symptoms or signs, particularly in those with extra-cranial solid tumours, and when these relapses are detected by imaging, there is no increase in survival. This review aims to establish whether routine surveillance imaging is beneficial, as well as evaluating the potential negative impacts of surveillance programmes.

**Systematic review registration:**

PROSPERO CRD42018103764

**Electronic supplementary material:**

The online version of this article (10.1186/s13643-019-1096-3) contains supplementary material, which is available to authorized users.

## Background

The follow-up of children and young people who have been treated for extra-cranial solid tumours currently involves a series of clinical reviews, along with imaging studies such as chest X-rays, CT or MRI scans or nuclear imaging, the exact nature of which is tailored to the original disease and directed by local protocols and traditions. The aim of providing regular imaging in this off-treatment phase is to detect recurrence of disease before clinical symptoms and signs develop and to establish any long-term complications of therapy [1].

The rationale for routine imaging for this group relies on a number of unproven assumptions [2]. The first of these is that this imaging will detect recurrence of cancer before the child develops clinical symptoms of the disease. The second is that because of this earlier detection, there will be treatment options available that would not be available later or would be more effective if given earlier. Finally, and most importantly, is the assumption that detecting a relapse at an earlier stage will prevent the child from dying of their cancer. Unfortunately, this may not be the case. Some studies have suggested that most relapses of childhood cancer are detected due to clinical symptoms or signs, particularly in those with extra-cranial solid tumours, and when these relapses are detected by imaging, there is no increase in survival [1, 3, 4]. However, as relapsed disease in children is thankfully rare, there are very small studies which have not been powered to detect even relatively large differences in survival of over 10% [3].

The answer to this systematic review’s main question is important to establish as routine imaging programmes carry a number of disadvantages. There is growing evidence that even relatively small amounts of radiation, such as that involved in a single CT scan, can significantly increase the risk of malignancy [2]. In children who may already be predisposed to developing cancer, performing additional CT scans may provide long-term risks. For very young children who require repeated general anaesthetics for their imaging, there are additional risks to their health. Furthermore, many families describe that routine follow-up imaging causes anxiety and distress, particularly whilst awaiting results. This may be avoided if routine imaging was found to be unnecessary. An additional disadvantage to regular imaging is the risk of false-positive findings, which result in unnecessary tests, treatment and further distress for the patient and family [4].

Finally, routine imaging programmes involve a number of costs. Families may incur transport and parking costs, as well as loss of work productivity, due to additional visits to the hospital. For the hospital itself, there are the costs of performing and interpreting the scans, as well as the staff and resource time in using imaging departments for this purpose. The opportunity costs may be marked when considered in terms of the number of images involved.

We therefore propose that a systematic review aiming to gather together all studies that have ever addressed the question of routine imaging may reach the numbers of patients necessary to provide a more precise answer to whether surveillance imaging is beneficial in terms of survival, in all children or in specific tumour groups. As far as we are aware, no such systematic review has previously been performed for this population group. For the purpose of this review, the term “child” or “childhood” refers to any child, adolescent or young adult up to the age of 25 years.

## Patient and public involvement group

The patient and public involvement (PPI) group for this research was a mixed group of young people who had been treated for a childhood cancer and parents of children who had been treated. Some parents had experience of a relapse of their child’s cancer, and some of their children had died. Some of the people who had had cancer had been treated as young children, and some had been treated as teenagers. The cancers experienced by the PPI group included neuroblastoma, clear cell sarcoma of the kidney, germ cell tumour, Ewing’s sarcoma and retinoblastoma. The group was invited through parent organisations (including the Children’s Cancer and Leukaemia Group (CCLG), Candlelighters Children’s Cancer Charity and Paediatric Oncology Reference Team (PORT)) as well as through Twitter and Facebook groups. Different members of the group had different social backgrounds and experiences. Three members of the group met with JM and RSP at a project inception event, in June 2018. Other members of the group provided input via telephone conversations with JM before or after the group.

Ultimately, the PPI group input has significantly changed the focus and design of this research, particularly changing the balance of the work to include more of the psychological and experiential aspects of surveillance imaging. Firstly, we recognised a need to change the title of the work to capture more accurately the concerns of these key stakeholders—whilst survival is important, it is not the only outcome to be considered. Therefore, the review title now captures the aims of establishing both benefits and harms of routine surveillance imaging.

Secondly, the PPI group has helped to create an order of priority for the work, including what research should be recommended if gaps appear in the current literature. All group members felt that the survival question was the first factor to explore and this informs all the following issues. We are also aware that the survival question is the area that is most likely to have sufficient data within the existing literature. Following on from the survival question, the anxiety and certainty issues were felt to be the next most important questions to answer within this review. We are aware the psychological health impacts may be less robustly researched.

Thirdly, the group has helped to focus the team’s attention psychological outcomes, as well as the role of timing and social setting on the experience of surveillance imaging. Two outcomes that we had not initially planned to include in the review, but which now form part of the protocol, are the rate of detection of “indeterminate” findings and the number of imaging tests performed that do not form part of the routine surveillance (so as to try to answer the issue of whether surveillance imaging reduces imaging exposure). We will now be seeking a broader range of research for inclusion within the review, including qualitative and survey data that attempts to capture experiences of surveillance imaging following childhood cancer alongside the quantitative data surrounding survival and costs.

Finally, the PPI group has helped to shape and clarify a dissemination plan for the research—emphasising the importance of certain stakeholders. The whole group has shared their keenness to continue to be involved in the work of this project, and we appreciate their input.

## Aims, objectives and overview of systematic review

To systemically review, critically appraise and synthesise the current literature surrounding off-therapy surveillance imaging in children and young people with extra-cranial solid tumours, in high-income countries, with a view to establishing if routine imaging studies after treatment for childhood cancer:

- Increase overall survival
- Increase the psychological distress caused to patients and families
- Result in other harms to children and young people
- Are cost-effective strategies

## Methods

This protocol is presented according to the PRISMA-P guidelines for the presentation of systematic review protocols (see Additional file 1) [5]. The work has been registered at PROSPERO ref: CRD42018103764.

### Search and retrieval strategy

The following databases will be searched to identify relevant studies: MEDLINE (Epub Ahead of Print, In-Process & Other Non-Indexed Citations, Ovid MEDLINE Daily and Ovid MEDLINE), PubMed, EMBASE, PsycINFO, Cumulative Index to Nursing & Allied Health (CINAHL Plus), Science Citation Index, Conference Proceedings Citation Index – Science, Cochrane Database of Systematic Reviews (CDSR), Cochrane Central Register of Controlled Trials (CENTRAL), Health Technology Assessment database, Database of Abstracts of Reviews of Effects, NHS Economic Evaluation Database (NHS EED) and EconLit. The search strategy for MEDLINE has been developed by an information specialist (MH) with input from the review team. The strategy comprises of subject headings and free-text terms for children/young people, cancer, imaging tests and surveillance (see Additional file 2). The MEDLINE strategy will be adapted as necessary for the other sources searched.

Conference proceedings of the RCPCH (Royal College of Paediatrics and Child Health), SIOP (International Society of Paediatric Oncology), ASPHO (American Society of Pediatric Hematology/Oncology), ASCO (American Society of Clinical Oncology) and ASH (American Society of Hematology) meetings will be searched for relevant abstracts. ClinicalTrials.gov and the WHO International Clinical Trials Registry Platform portal will be searched for relevant ongoing work. Reference lists of relevant systematic reviews and included articles will also be reviewed. Forward citation searching of included articles will be performed, using Web of Science. Authors of relevant studies may be contacted as time allows to seek further studies. Published and unpublished studies will be sought and no language, geographical or study design restrictions applied. Non-English language studies will be translated if time permits. Searches will be limited to studies from 1990 onwards, to reflect the current era of survival in childhood cancer.

### Screening for eligibility

Screening and data extraction will be managed using EPPI-Reviewer 4 [6]. Two reviewers (JM and another researcher) will independently screen the title and abstract of studies for inclusion. After each 10% portion of the records has been screened, the rate of agreement between reviewers will be assessed. Once an adequate rate of agreement has been reached (> 90% agreement), the remaining records will be split between the two reviewers and single screened. Full texts of studies which might be relevant will then be sought and assessed further, using the study eligibility decision form (Appendix 1). Full-text screening will be performed by two independent researchers. Disagreements will be resolved by consensus or, if this proves impossible, by recourse to an independent adjudicator (RSP).

### Inclusion and exclusion criteria for quantitative studies

#### Study design

The study designs to be included in the review will be added in an iterative way. Initially, we will use randomised controlled trials (RCTs) and quasi-randomised trials to attempt to answer the research aims. If, as we suspect, there are insufficient studies of these designs, we will then move to include prospective cohort studies and re-evaluate the data. Finally, should the research objectives not be met using these study designs, we will then go on to consider the inclusion of retrospective studies. Surveys of patient or provider opinions will not be actively sought in this review, but when identified will be included within the narrative review as they may provide insight into experiences and priorities that are not found elsewhere. Case studies will not be eligible for inclusion within the review. We acknowledge the progressive increase in risk of bias as we move through this strategy, and these challenges will be discussed within our reports and publications.

#### Population

The study will include children or young people up to age 25 years who have completed treatment for a malignant extracranial solid tumour and have no evidence of active and ongoing disease at the end of treatment. This may include patients with residual abnormalities which are deemed to be stable at the time of entry to the study. Tumour types include but are not limited to neuroblastoma, Wilms’ tumour, soft tissue sarcoma (including rhabdomyosarcoma), Hodgkin’s lymphoma, malignant bone tumours (including osteosarcoma and Ewing’s sarcoma) and extra-cranial germ cell tumours.

Studies where the majority (> 50%) of patients are aged less than 25 years will be included, even if children and young people are not reported separately, particularly if the tumour type is reported (for example, neuroblastoma). If studies report a mixed population but data related to children and young people can be extracted separately to older adults will be included.

Studies which evaluate screening programmes solely related to the development of malignancies in patients with cancer predisposition syndromes will not be eligible for inclusion as these strategies have different aims and objectives (namely detecting new-onset primary malignancies rather than relapse).

We will not include studies from low- and middle-income countries (LMICs) given the differences in disease presentation, management and risk of relapse within these settings, which may result in different risks and benefits from routine surveillance imaging. Given that the results of this review are to be applied within the HIC setting, we have specifically focused on studies performed here.

#### Interventions and comparators

We are aware that studies are likely to include a wide range of surveillance imaging strategies. For the purpose of this review, studies must evaluate a programme of surveillance imaging that aims to detect a relapse of previously treated childhood cancer, at the site of previous disease or likely metastatic recurrence. Studies of surveillance imaging programmes looking predominantly for late effects of treatment will not be eligible for inclusion as these form a different set of aims and objectives and lie outwith the remit of this review. Non-randomised and single-arm studies must examine the surveillance imaging programme as the primary aim of the study report. We anticipate that many of these studies will be performed as secondary studies running alongside larger trials of upfront or relapse treatment options.

The surveillance imaging programme must include some form of radiological imaging, including (but not limited to) X-ray, ultrasound and cross-sectional or nuclear imaging techniques.

Comparator groups within RCTs or cohort studies will include routine follow-up without radiological imaging, which result in “symptom or sign” based detection of relapsed disease. Surveillance programmes which involve routine clinical review and examination, including examinations under anaesthesia (EUAs) for retinoblastoma, will therefore form a comparator group rather than an intervention. Studies comparing two different surveillance imaging programmes will be eligible for inclusion.

Studies without a comparison group are eligible for inclusion, provided that they meet all other inclusion criteria.

#### Outcomes

The selection of outcomes has been substantially informed by the work with the PPI group for this research. The group was very clear that overall survival was the primary outcome for the review, with other secondary outcomes being identified as listed.

Primary outcome:

- Overall survival (OS)—evaluated as age at the time of death, or from date of original diagnosis. This may be defined differently by each study, and therefore, alternative definitions may be considered. Importantly, studies reporting only survival from diagnosis of relapse will not be included as this is likely to be dependent upon the methods used for detection of relapse and subsequently at risk of lead-time bias.

Secondary outcomes:

- Psychological distress indicators—anxiety scores and quality of life (QoL) scores, assessed over different groups, including children, teenagers, parents and other family members (such as siblings and grandparents)
- Other harms of imaging—including but not limited to general anaesthetics required, second malignancies, side effects of sedation and “indeterminate” findings
- Number of imaging tests performed as part of surveillance programme and number of imaging tests performed not as part of surveillance programme
- Cost-effectiveness measures, including diagnostic yield per investigation

### Inclusion and exclusion criteria for qualitative studies

Studies will be eligible for inclusion if they meet all of the following criteria:

#### Study design

All studies using qualitative methodology will be eligible for inclusion, including but not limited to ethnography, phenomenology and grounded theory. Studies that use qualitative methods but which do not state an explicit methodology are also eligible to be included, provided that they present qualitative data. This includes, but is not limited to, studies using focus group discussions, interview studies and observational studies. Similarly, mixed methods studies are eligible for inclusion if they provided sufficient data.

#### Study participants

The study participants will include patients, their parents/carers, healthcare professionals, commissioners and/or policy makers—though we anticipate that any available data is most likely to have consulted parents, and occasionally patients. We do not anticipate finding any qualitative work of healthcare professionals, commissioners and/or policy makers, but if this is present, it will be eligible for inclusion. The topic of interest explored should be surveillance imaging following treatment for paediatric extra-cranial solid tumours.

#### Outcome of interest

Experiences of surveillance imaging.

#### Language

Qualitative data studies will be limited to those performed and written in the English language. The benefit of qualitative research is to allow participants to express their experiences, the clarity of which could be lost through translation and thus the results of the synthesis may less accurately capture the views of participants.

### Data extraction

Data will be extracted by one researcher using a standardised data extraction form and independently checked by a second (see Appendix 2 for the planned data variables). In addition, for studies describing categorical test information, information will be extracted on any cut points used (with the technique used for derivation of cut points) and methods of statistical analysis, including variables adjusted for. If the data to be extracted is unclear, the corresponding author will be contacted for further information. If there is no response, a further attempt to make contact will be made a fortnight later. If there is no response after a further 4 weeks, the data will be presumed unavailable.

### Assessment of risk of bias

The quality of studies will be assessed at outcome level using a stratified version of the ROBINS-I tool, supplemented with information about potential sources of heterogeneity: patient demographic and clinical characteristics, study era, geography and antibiotic use [7, 8]. A stratified version of the ROBINS-I tool is justified given the significant increase in resources required for full use of the tool. Instead, we will perform a simplified version of the tool for all studies (see Appendix 3) and only proceed to a full ROBINS-I assessment with studies which are considered to be at low to moderate risk of bias.

### Methods of analysis/synthesis

Key study characteristics, the outcome data and study quality will be summarised in narrative and tabular forms. A mapping phase will be performed for the review, clearly laying out the studies (and included data) according to the relevant cancer type, imaging modality, timings of surveillance and study type. Analysis beyond these descriptive steps will take an iterative approach dependent upon the studies identified and data available from these. We anticipate that minimal statistical analysis will be possible given the likely heterogeneity of the included data. Should they be possible, the following analytical steps will be taken.

#### Narrative analyses

Narrative synthesis of the quantitative data will focus on the features of each surveillance programme reported and seek to identify key themes within the outcomes, taking into account the assessments of risk of bias. The narrative analysis will be split according to cancer type. For each cancer type, data will be reported of number of studies, patients and relapses included. Where reported, we will summarise how relapses were diagnosed (by surveillance or by symptoms). We will report any survival data presented by the studies. If reported by the studies, we will then present any data on the number of images performed (including radiation dose received) and any cost-effectiveness, qualitative or psychological distress indicator data.

#### Meta-analysis

Where appropriate, data will be synthesised using a random effects meta-analysis using the R programming environment [9]. The meta-analysis will be based on ratio measures or survival duration, if provided, and only if sufficient clinical homogeneity exists. Inverse variance random effects meta-analysis will be used given the anticipated clinical heterogeneity in terms of population and intervention.

Heterogeneity will be explored both clinically and statistically. Clinical evaluation of heterogeneity will consider the differences in the surveillance programmes assessed, different tumour types and ages of patient included in the study and other factors such as healthcare service design. Statistical heterogeneity will be examined using *χ*^2^ tests, the *I*^2^ and tau^2^ statistics and by visual inspection of the forest plots.

#### Subgroup analyses

Subgroup analyses and sensitivity analyses will be performed, including but not limited to tumour type, presence residual tumour, imaging techniques, timing and duration of the surveillance imaging and type of health care service.

##### Tumour type

Studies will be analysed separately by tumour type, given that the benefits of a screening programme such as routine surveillance imaging depend upon there being an effective intervention for relapse, with intervention at a pre-symptomatic phase leading to better outcomes [10]. The likelihood of such an intervention varies between tumour types and thus has a significant impact of the usefulness of surveillance imaging.

##### No evidence of disease vs stable residuals

Where possible, studies, or subgroups within studies, will be analysed according to the status of patients’ disease at the start of routine surveillance imaging. Patients with stable residual disease might be considered at increased risk of relapse compared to those with no evidence of disease and as such might be more likely to benefit from routine surveillance imaging to identify this.

##### Imaging technique

Studies using different imaging techniques will be analysed separately given that the different modalities may have different diagnostic test accuracies for identifying relapse and thus may identify relapse at different stages. Thus, imaging with one modality may be more or less effective as a routine surveillance programme than another, even within the same tumour type.

##### Timing of surveillance (i.e. length of intervals)

The frequency of surveillance imaging is likely to impact on the risk of length time bias and thus impact on the survival benefits of the surveillance programme. It may also inversely impact on psychological outcomes, with frequent scans increasing the acuity of the sawtooth mood variation of “scanxiety”. The frequency will be grouped by number of months between scans, recognising that most programmes will use 3-, 4-, or 6-monthly imaging particularly in the early phases after treatment.

##### Duration of surveillance (i.e. time from start to end of surveillance programme)

The duration of surveillance imaging may impact upon the psychological impacts, cost-effectiveness and other potential disadvantages of screening programmes. This subgroup will be evaluated by grouping the duration of imaging into 6-monthly blocks from the start of the programme.

##### Type of healthcare service

The type of healthcare service (public or private) may impact upon the costs of imaging, as well as attitudes towards surveillance imaging for professionals and families. The subgroup will evaluate any cost data according to public or private healthcare.

#### Sensitivity analyses

Potential areas of heterogeneity will be explored using sensitivity analyses, including study design (including restricting analyses to randomised controlled trials only), studies reported as conference abstracts only, risk of bias assessed by components, definitions of overall survival (age at death vs OS from original diagnosis vs other) and the location of the study, which provides information on the surrounding healthcare system.

We will also explore whether the original treatment programme for the malignancy has an impact on relapse detection, as well as overall survival. In the situation of different original treatment programmes, the ability of the relapse to be salvaged is likely to change and directly affect survival. Additionally, different original treatment programmes may affect the speed of development, or location, or tracer-uptake characteristics of relapse; this may lead to a difference in the ability of the imaging programme to detect relapse occurring.

#### Publication bias

The risk of publication bias will be explored if there are ≥ 5 comparative studies reporting the same outcome using contour-enhanced funnel plots and Harbord and Peters tests [7]. We anticipate that the risk of publication bias in this field will be relatively large, as studies are likely to be small and performed as secondary analyses within larger studies (thus showing a lag time bias).

#### Qualitative analysis

Any qualitative data will be analysed using thematic analysis to combine data relating to perceived risks and benefits of routine surveillance imaging, and experiences related to the routine surveillance imaging process, separated according to stakeholder group (patient, family, professional, etc.). The analysis will explore the impact of different methods of data collection, the patient’s outcome (those who experienced relapse compared with those who did not), tumour type and the different features of the routine surveillance imaging programme (imaging type, timing, duration and healthcare service setting). Coding will be independently performed by two researchers and then discussed within the research team.

### Methods of dissemination

The dissemination plan for this review has been developed alongside our PPI group. The dissemination plan will need to vary dependent upon the findings of the research, with emphasis on different stakeholders being informed dependent upon the certainty of the findings and the likely impact of these. Dissemination will include traditional methods including journal manuscripts, conference posters and presentations, with data reported according to PRISMA guidelines [11]. Simultaneously, we will seek to disseminate results to patients, families, healthcare professionals, researchers and research funders through methods such as infographics of key findings, promotional videos, social media updates and presentations. The study team (researchers and PPI group) feels that one of the most important aspects of the dissemination plan is to provide a layered approach to allow people to access as much or as little information as they would like about the research.

## Discussion

This review has one of three potential outcomes: (1) that routine surveillance imaging conveys a survival benefit, (2) that routine surveillance imaging does not convey a survival benefit or (3) that there is insufficient evidence to answer the research question. The impact of each of these potential outcomes is discussed below.

If the review finds that routine surveillance imaging is beneficial, it will provide information on the groups of patients who may benefit, and may also be able to comment on the optimal timing and duration of imaging, dependent upon the evidence available.

If this systematic review finds that routine surveillance imaging does not convey a survival benefit to children and young people with extra-cranial solid tumours, this could have significant benefits. As discussed above, reducing exposure to radiation, anaesthesia, scan anxiety and risks of false-positive results could dramatically improve the current patient experience and future health of survivors of childhood cancer, whilst also conveying financial and service benefits. Should this be the case, the work has the potential to impact clinical practice rapidly, with the systematic review process being relatively fast compared to primary research and implementation of the work requiring minimal further costs after the establishment of the results.

If the review finds that there is insufficient evidence to address the questions surrounding routine surveillance imaging, it will have identified the current gaps in the literature and be able to define the design of future studies that are most likely to provide the evidence needed.

### Additional files

Additional file 1: PRISMA-P guidelines. (DOCX 33 kb)

Additional file 2: Search strategy. (DOCX 14 kb)

## Data Availability

Not applicable (all data that is referred to in this article will have been obtained through reading the original articles or contacting the authors of cited studies).

## Appendix 1

### Study eligibility decision form

Person completing form:

Title of study:

Authors of study:

1. Does the study include ≥ 50% children or young adults aged less than 25 years (or is data for this group extractable)?

Yes/Unclear/No

2. Does the study include patients who have completed treatment for a malignant extracranial solid tumour and have no evidence of active and ongoing disease at end of treatment?

Yes/Unclear/No

3. Does the study include patients treated in a high income setting (or is the data for this group extractable)?

Yes/Unclear/No

4. Does the study examine routine surveillance imaging outcomes (with or without a control/comparisor group)?

Yes/Unclear/No

5. Does the study assess any of the outcomes defined in the review protocol?

Yes/Unclear/No

6. Final decision (Include only if all 5 previous questions answered Yes, Exclude if any No.): Include/Exclude/Unsure
7. If exclude, main reason for exclusion:

## Appendix 2

### Data extraction tool

#### General information

Person performing data extraction:

Date of data extraction:

Study title:

Study Author, Year:

Language:

Country (or countries) in which research was performed:

Source of funding:

#### Study Information

Stated aim of study:

Study design:

Appropriate risk of bias tool completed: ☐

Inclusion criteria:

Exclusion criteria:

Definition of no evidence of active or ongoing disease:

Routine imaging used (please complete information for each imaging modality separately):

Imaging modality: X-ray/ultrasound/CT/MRI/bone scan/other

Site of imaging (e.g. affected site, abdomen, chest):

Frequency of imaging (e.g. every 6 months):

Duration of imaging programme (e.g. over 5 years):

Details of reporting/quality control:

Comparator/control group (if present):

Details of randomisation/selection of cohorts:

#### Participants

Number of participants:

Number in each group:

Number withdrawn:

Number included in analysis:

Age – provide details for each group:

Sex – provide details for each group:

Ethnicity (if given):

Socio-economic status (if given):

Disease(s):

Other important population factors (e.g. any patients with hereditary predisposition syndromes):

Are recruitment/refusal to consent numbers given? If so, please record details including, if given, number, distribution, reasons for declining:

#### Outcomes

Definition of relapse used:

Primary outcome(s), including definition of each:

Secondary outcome(s), including definition of each:

## Appendix 3

### Assessment of risk of bias – stratified approach to ROBINS-I

#### First stage assessment

1) Is there potential for confounding of the effect of intervention in this study?

2) Was selection of participants into the study (or analysis) based on participant characteristics observed after the start of intervention?

3a) Were there deviations from the intended surveillance programme or control arm beyond expected in usual practice?

3b) Were these unbalanced and likely to have affected outcome?

4a) Were outcome data available for all, or nearly all, participants?

4b) Is there evidence that results were robust to the presence of missing data?

5a) Could the outcome measure have been influenced by knowledge of the intervention received?

5b) Were outcome assessors aware of the intervention received by study participants?

5c) Were any systematic errors in measurement of the outcome related to intervention received? (e.g. survival measured from diagnosis of relapse)

6) Is the reported effect estimate likely to be selected on the basis of the results from multiple outcome measurements within the outcome domain, multiple analyses of the intervention-outcome relationship or different subgroups?

#### Second stage assessment

Full ROBINS-I tool as published [8].

## Abbreviations

ASCO: American Society of Clinical Oncology
ASH: American Society of Hematology
ASPHO: American Society of Pediatric Hematology/Oncology
CCLG: Children’s Cancer and Leukaemia Group
CT: Computerised tomography
EUA: Examination under anaesthesia
LMICs: Low- and middle-income countries
MRI: Magnetic resonance imaging
PORT: Paediatric Oncology Reference Team
PPI: Patient and public involvement
PRISMA: Preferred Reporting Items for Systematic Reviews and Meta-Analyses
PRISMA-P: Preferred Reporting Items for Systematic review and Meta-Analysis Protocols
QoL: Quality of life
RCPCH: Royal College of Paediatrics and Child Health
RCTs: Randomised controlled trials
SIOP: International Society of Paediatric Oncology
