# Supplementary material for: Does routine surveillance imaging after completing treatment for childhood solid tumours cause more harm than good? A systematic review and meta-analysis protocol
Source: Syst Rev. 2019 Jul 12;8:168. doi: 10.1186/s13643-019-1096-3 (PMC6624999; doi:10.1186/s13643-019-1096-3)
Supplement: Supplementary file 2 — Search strategy. (DOCX 14 kb) [file 13643_2019_1096_MOESM2_ESM.docx]

Database: Ovid MEDLINE(R) and Epub Ahead of Print, In-Process & Other Non-Indexed Citations, and Daily <1946 to July 18, 2018>

19^th^ July 2018

Search Strategy:

--------------------------------------------------------------------------------

1 exp Child/ (1778058)

2 Adolescent/ (1873153)

3 exp Infant/ (1069283)

4 Young Adult/ (672935)

5 (child$ or infant$ or infancy or pediat$ or paediat$ or preschool$ or pre school$ or schoolchild$ or school age$ or schoolage$ or schoolboy$ or schoolgirl$).ti,ab. (1655832)

6 (girl or girls or boy or boys or kid or kids).ti,ab. (214747)

7 (adoles$ or puberty or prepuberty or pubescen$ or prepubescen$ or teen$ or youth$ or preteen$ or juvenil$).ti,ab. (383924)

8 (neonat$ or neo nat$).ti,ab. (239450)

9 (newborn$ or new born$ or newly born$).ti,ab. (154032)

10 (baby or babies).ti,ab. (63955)

11 (young adj2 (adult or adults or person or persons or people)).ti,ab. (106954)

12 or/1-11 (4282554)

13 exp Neoplasms/ (3060910)

14 (cancer$ or neoplas$ or carcinoma$ or adenocarcinoma$ or tumour$ or tumor$ or oncolog$).ti,ab. (2807200)

15 (malignan$ or metastas$ or metastat$).ti,ab. (857209)

16 (lymphoma$ or sarcoma$ or Hodgkin$ or non-Hodgkin$).ti,ab. (258471)

17 (angiosarcoma$ or hemangiosarcoma$ or carcinosarcoma$).ti,ab. (10184)

18 (reticulolymphosarcoma$ or germinoblastoma$ or immunoblastoma$ or lymphogranuloma$ or lymphosarcoma$ or reticulosarcoma$ or lymphomatoid granulomatosis).ti,ab. (9555)

19 (blastoma$ or neuroblastoma$ or esthesioneuroblastoma$ or ganglioneuroblastoma$).ti,ab. (35466)

20 (osteosarcoma$ or Ewing$).ti,ab. (27927)

21 Rhabdomyosarcoma$.ti,ab. (10974)

22 (Wilm$ or nephroblastoma$).ti,ab. (11401)

23 (hepatoma$ or hepatoblastoma$).ti,ab. (30022)

24 retinoblastoma$.ti,ab. (15231)

25 (teratocarcinoma$ or teratoma$ or dysembryoma$ or dermoid cyst$ or dermoids or struma ovarii).ti,ab. (19969)

26 (chordoma$ or chordocarcinoma$ or chordoepithelioma$ or notochordoma$).ti,ab. (3551)

27 (germinoma$ or dysgerminoma$ or nonseminoma$ or seminoma$).ti,ab. (10877)

28 (gonadoblastoma$ or mesonephroma$ or choriocarcinoma$ or neuroepithelioma$).ti,ab. (7317)

29 (melanoameloblastoma$ or melanotic progonoma$ or adenoma$ or apudoma$ or melanoma$).ti,ab. (181671)

30 or/13-29 (3974767)

31 exp Diagnostic Imaging/ (2442114)

32 (imag$ adj6 (diagnos$ or test$ or tool$ or procedure$ or protocol$ or method$ or technique$ or technolog$ or modalit$ or mode or modes)).ti,ab. (271356)

33 (radiolog$ adj3 (diagnos$ or test$ or tool$ or procedure$ or protocol$ or method$ or technique$ or technolog$ or modalit$ or mode or modes)).ti,ab. (28446)

34 (radionuclide adj3 (diagnos$ or test$ or tool$ or procedure$ or protocol$ or method$ or technique$ or technolog$ or modalit$ or mode or modes)).ti,ab. (2944)

35 radiograph$.ti,ab. (193645)

36 (x-ray$ or xray$ or roentgen$).ti,ab. (346461)

37 (bone$ adj2 (scan$ or imag$)).ti,ab. (12277)

38 (radiolog$ adj2 (scan$ or imag$ or assessment$)).ti,ab. (11658)

39 (radionuclide adj2 (imag$ or scan$ or assessment$)).ti,ab. (4911)

40 (radioisotope$ adj2 (scan$ or imag$ or assessment$ or diagnos$)).ti,ab. (1442)

41 (nuclear adj2 (medicine or imag$ or scan$)).ti,ab. (14900)

42 ((magnetic resonance adj (imag$ or scan$ or tomograph$)) or MRI or MR imag$ or MR scan$ or MR tomograph$ or MRT or NMR or NMRI or fMRI or chemical shift imag$).ti,ab. (528899)

43 ((compute$ adj2 tomograph$) or tomodensitometry or cine-CT).ti,ab. (261941)

44 ((CT or CAT) adj (scan$ or imag$)).ti,ab. (107831)

45 (cross-sectional adj2 (scan$ or imag$)).ti,ab. (6176)

46 ((emission or positron or proton) adj2 tomograph$).ti,ab. (66798)

47 (PET or PET-CT$ or PET?CT$ or CT-PET$ or CT?PET$).ti,ab. (82799)

48 (SPECT or SPECT-CT$ or SPECT?CT$ or CT-SPECT$ or CT?SPECT$).ti,ab. (25933)

49 (SPET or SPET-CT$ or SPET?CT$ or CT-SPET$ or CT?SPET$).ti,ab. (1324)

50 (PET-MRI$ or PET?MRI$).ti,ab. (1379)

51 Fluorodeoxyglucose F18/ (26044)

52 ((FDG or fluorodeoxyglucose) adj4 (imag$ or scan$)).ti,ab. (10023)

53 (FDG-PET$ or FDG?PET$).ti,ab. (21270)

54 (ultrasound$ or ultrasonograph$ or echograph$ or ultrasonic$ or sonograph$ or echotomograph$ or echogram$ or echoscop$ or echosound$).ti,ab. (364789)

55 or/31-54 (3291471)

56 12 and 30 and 55 (120353)

57 Population Surveillance/ (54799)

58 surveil$.ti,ab. (149998)

59 ((follow-up$ or followup$ or followed up) adj6 (assess$ or monitor$ or review$ or investigation$ or screen$)).ti,ab. (54321)

60 ((follow-up$ or followup$ or followed up) adj6 (routine$ or regular$ or periodic$ or long-term or longer-term or frequen$ or repeat$ or repetitive$)).ti,ab. (84384)

61 ((follow-up$ or followup$ or followed up) adj6 (off-therapy or off-treatment or post-therapy or posttherapy or post-treatment or posttreatment)).ti,ab. (4135)

62 ((follow-up$ or followup$ or followed up) adj3 (duration$ or timing$ or schedul$ or serial$ or optimal$ or optimum or intensive$ or intensity or interval$)).ti,ab. (29247)

63 ((follow-up$ or followup$ or followed up) adj3 (clinical or protocol$ or strateg$ or guideline$ or recommend$ or standard$ or program$ or policy or policies)).ti,ab. (43400)

64 ((routine$ or regular$ or periodic$ or long-term or longer-term or repeat$ or repetitive$ or frequen$) adj3 (assess$ or monitor$ or review$ or investigation$ or screen$)).ti,ab. (105749)

65 ((off-therapy or off-treatment or post-therapy or posttherapy or post-treatment or posttreatment) adj3 (assess$ or monitor$ or review$ or investigation$ or screen$)).ti,ab. (2301)

66 or/57-65 (467619)

67 56 and 66 (6964)

68 (surveil$ adj4 (imag$ or scan$ or radiolog$ or radiograph$)).ti,ab. (2307)

69 ((follow-up$ or followup$ or followed up) adj3 (imag$ or scan$ or radiolog$ or radiograph$)).ti,ab. (21677)

70 ((routine$ or regular$ or periodic$ or long-term or longer-term$ or repeat$ or repetitive$ or frequen$) adj3 (imag$ or scan$ or radiolog$ or radiograph$)).ti,ab. (29491)

71 ((off-therapy or off-treatment or post-therapy or posttherapy or post-treatment or posttreatment) adj4 (imag$ or scan$ or radiolog$ or radiograph$)).ti,ab. (1879)

72 (serial$ adj2 (imag$ or scan$ or radiolog$ or radiograph$)).ti,ab. (6041)

73 or/68-72 (58224)

74 12 and 30 and 73 (4276)

75 67 or 74 (9949)

76 exp animals/ not humans/ (4476555)

77 75 not 76 (9936)

78 limit 77 to yr="1990 -Current" (9201)
